# Computer quantification of airway collapse on forced expiration to predict the presence of emphysema

**DOI:** 10.1186/1465-9921-14-131

**Published:** 2013-11-19

**Authors:** Marko Topalovic, Vasileios Exadaktylos, Anneleen Peeters, Johan Coolen, Walter Dewever, Martijn Hemeryck, Pieter Slagmolen, Karl Janssens, Daniel Berckmans, Marc Decramer, Wim Janssens

**Affiliations:** 1 Respiratory Division, University Hospital Leuven, Department of Clinical and Experimental Medicine, KU Leuven, Leuven, Belgium; 2 Department of Biosystems, Measure, Model and Manage Bioresponses (M3 BIORES), KU Leuven, Leuven, Belgium; 3 Department of Radiology, University Hospital Leuven, Leuven, Belgium; 4 Department of Electrical Engineering, Medical Image Computing, ESAT/PSI, KU Leuven, Leuven, Belgium; 5 iMinds – KU Leuven Future Health Department, Leuven, Belgium; 6 Medical Imaging Research Center, KU Leuven & UZ Leuven, Leuven, Belgium; 7 Research and Development, LMS International, Leuven, Belgium

**Keywords:** Spirometry, Pulmonary emphysema, Flow-volume loops, Chronic obstructive pulmonary disease, Lung collapse

## Abstract

**Background:**

Spirometric parameters are the mainstay for diagnosis of COPD, but cannot distinguish airway obstruction from emphysema. We aimed to develop a computer model that quantifies airway collapse on forced expiratory flow–volume loops. We then explored and validated the relationship of airway collapse with computed tomography (CT) diagnosed emphysema in two large independent cohorts.

**Methods:**

A computer model was developed in 513 Caucasian individuals with ≥15 pack-years who performed spirometry, diffusion capacity and CT scans to quantify emphysema presence. The model computed the two best fitting regression lines on the expiratory phase of the flow-volume loop and calculated the angle between them. The collapse was expressed as an Angle of collapse (AC) which was then correlated with the presence of emphysema. Findings were validated in an independent group of 340 individuals.

**Results:**

AC in emphysema subjects (N = 251) was significantly lower (131° ± 14°) compared to AC in subjects without emphysema (N = 223), (152° ± 10°) (p < 0.0001). Multivariate regression analysis revealed AC as best indicator of visually scored emphysema (R^2^ = 0.505, p < 0.0001) with little significant contribution of K_CO,_ %predicted and FEV_1,_ %predicted to the total model (total R^2^ = 0.626, p < 0.0001). Similar associations were obtained when using CT-automated density scores for emphysema assessment. Receiver operating characteristic (ROC) curves pointed to 131° as the best cut-off for emphysema (95.5% positive predictive value, 97% specificity and 51% sensitivity). Validation in a second group confirmed the significant difference in mean AC between emphysema and non-emphysema subjects. When applying the 131° cut-off, a positive predictive value of 95.6%, a specificity of 96% and a sensitivity of 59% were demonstrated.

**Conclusions:**

Airway collapse on forced expiration quantified by a computer model correlates with emphysema. An AC below 131° can be considered as a specific cut-off for predicting the presence of emphysema in heavy smokers.

## Background

Chronic Obstructive Pulmonary Disease (COPD) is characterized by airflow limitation that is not fully reversible, usually progressive and associated with an abnormal inflammatory response of the lung to noxious particles or gases, most often from cigarette smoke [1]. Being 4th leading cause of death, COPD is one of the major health challenges of the next decades, while the World Health Organization predicts that it will become the 3rd leading cause of death by 2030 [2-4]. Prevalence surveys indicate that up to almost one quarter of the adults aged 40 years and older may have mild airflow obstruction [5]. One of the challenges in such pandemic is to identify patients at risk for rapid deterioration and to develop diagnostic tools and specific treatment strategies which are directed to clinically important subgroups or particular phenotypes with poor outcome [6,7].

Emphysema may be considered as one of the oldest phenotypes in COPD. It is characterized by the loss of lung tissue leading to breakdown of alveolar walls and subsequent airway collapse during forced expiration. It has been demonstrated that emphysema is associated with lower body mass index and bone mineral density, reduced exercise capacity, impaired quality of life and higher BODE index compared to COPD patients with similar airflow obstruction without emphysema [8-12]. Emphysema is, independently of COPD, a strong risk factor for lung cancer [13,14] and CT screening may be most beneficial for this particular subgroup [13,15]. Additionally, subtypes of emphysema patients may be referred for endoscopic valve placement, bullectomy or even lung volume reduction surgery. As most common variables obtained by spirometry do not accurately reflect emphysema presence, the diagnosis is currently based on CT scan of the chest, which is expensive, not routinely available and therefore not imposed every time a new diagnosis of COPD is made. Decreased variables of diffusing capacity such as D_L,CO_ and K_CO_ are often used as a surrogate marker of emphysema, for instance in the differential diagnosis with asthma, because they basically reflect impaired oxygen uptake by the loss of alveoli typically observed when the parenchyma is destroyed [16,17]. Again, these measures need more advanced equipment restricting the analysis to respiratory physicians in secondary care. An alternative approach, however, may be found in the correct quantification of collapse during the forced expiratory phase of a flow-volume loop, the so-called ‘spirographic kink’. This kink, suddenly diminished expiratory flow, has been linked to emphysema in the past but the association has only been based on visual assessments in limited patient samples [18-20]. We therefore hypothesized that a standardized quantification of collapse may offer a more precise indication of emphysema presence, which would be of particular interest in primary care.

In the present study our objective was first to develop a mathematical model for precise computerized quantification of airway collapse on forced expiratory flow-volume loops. Secondly, we related these measures of airway collapse to CT-diagnosed emphysema in two large independent cohorts of smoking individuals.

## Methods

### Study subjects

To develop the computer model we included data of 513 individuals of the LEUVEN COPD cohort who performed complete pulmonary function testing at cohort entry (including post-bronchodilator spirometry and diffusing capacity) and of whom a computed tomography (CT) scan was available within 1 year of enrolment. All subjects were included between October 2007 and January 2009 at the University Hospital of Leuven (Belgium), as earlier described [21,22]. Briefly, participants were all current or former heavy smokers with at least 15 pack-years and with minimal age of 50 years. Individuals with suspicion or diagnosis of asthma were excluded, as well as patients with exacerbations due to COPD within last 6 weeks and patients with other respiratory diseases. To validate findings, a separate cohort of 340 individuals was recruited between January 2009 and November 2010 at the University Hospital of Leuven. Similar measurements, inclusion and exclusion criteria were applied. Study was approved by the local ethical committee of the University Hospital Leuven, (KU Leuven, Belgium). All patients included in the study provided informed consent. Study design of the LEUVEN COPD cohort can be found on http://www.clinicaltrials.gov (NCT00858520).

### Pulmonary function tests

All pulmonary function tests were performed with standardized equipment (Masterlab, Erich Jeager, Würzburg, Germany) by respiratory technicians, according to the ATS/ERS criteria [23]. Spirometry data are post-bronchodilator measures and expressed as percent predicted of normal reference values [24]. Diffusing capacity (D_L,CO_) was measured by the single-breath carbon monoxide gas transfer method and corrected for alveolar ventilation but not hemoglobin concentration [25]. Patients with COPD were identified when post-bronchodilator FEV1/FVC ratio was <0.7, based on international COPD guidelines [26].

### Emphysema scores

All CT scans were examined by two independent radiologists and visually scored for the presence and extent of emphysema at 3 predefined levels [21]. The presence of emphysema, which was defined as an area of hypovascular low attenuation, was graded at each level with increments of 5% and averaged as a percentage of total lung tissue over both lungs. The final scores were the mean percentages taken from scores of both radiologists, resulting in a linear variable ranging from 0 to 93.3%. Inter-observer variability determined with intraclass correlation coefficient (ICC) for the mean scores of the individual radiologists demonstrated strong agreement and consistency (ICC = 0.84, p < 0.0001). If emphysema was visually scored on any of the predefined fields, the patient was categorized as having emphysema. Additionally, quantification with CT lung densitometry was performed. The extent of emphysema was estimated using the percent of voxels with an X-ray attenuation value below -950 HU. A cut-off values of ≥1% and ≥10% of total lung volume attenuated below -950 HU was arbitrarily chosen as being abnormal and used to identify presence of emphysema [21,27]. A complete protocol for quantification of emphysema is available in the online supplement (Additional file 1: Note S1).

### Computer model

To develop a computer model for the calculation of angle of collapse (AC) we used MATLAB (7.14, The MathWorks, Natick, Massachusetts). In all individuals the best expiratory flow-volume curve (highest sum of FEV1 and FVC) within one spirometry was exported from the Masterlab system at a sampling rate of 125 Hz. By extracting data points it was possible to reconstruct the best expiratory manoeuvre in MATLAB and to develop a unique algorithm for automatic quantification of airway collapse (see Results section).

### Statistical analysis

Statistical analysis was performed using Statistical Analysis System (SAS) version 9.3, (SAS Institute, Cary, USA). The Shapiro-Wilk test was used to control normality of the datasets, while a *T*-test was used to evaluate differences in AC between patients with and without emphysema within disease severity stages. Linear-regression models were applied for continuous variables analyses and logistic-regression models for binary variables. Stepwise selection was used to identify the subset of variables that had the strongest relation to emphysema scores, using default criteria of significance at the 0.15 level to enter or egress the model. Receiver operating characteristic (ROC) curve analysis was performed with GraphPad Prism version 5.01, (GraphPad Software, La Jolla, California, USA).

## Results

### Computer model

**Figure 1:**
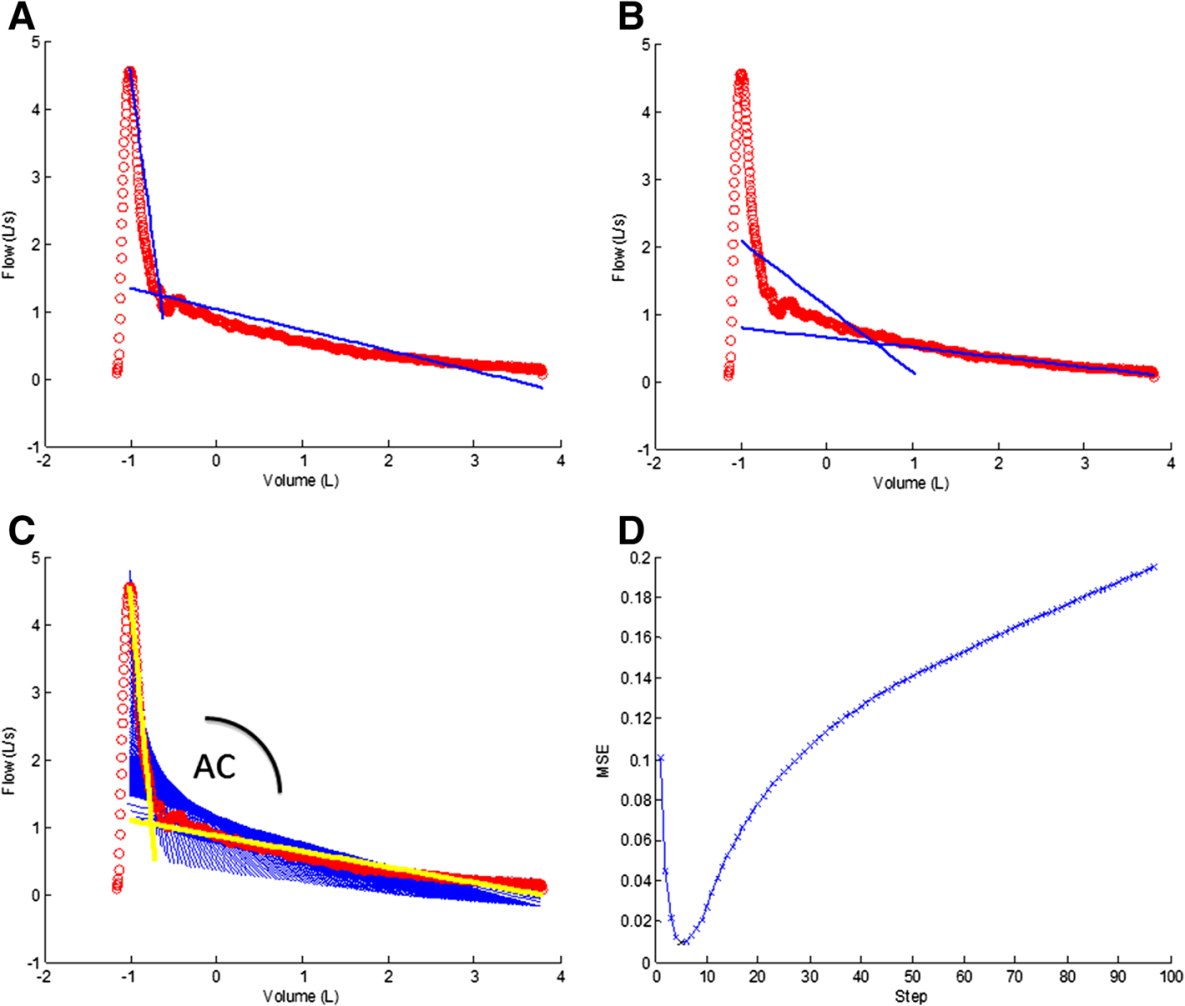
**Example of the calculation process with fitting of regression lines to the original data in incremental steps of 10 data points.** Panels **A-B** show regression lines for a candidate reference point after respectively 10 and 400 data points. Panel **C** shows all regression lines together with a highlighted selection of 2 lines in yellow (after 6 steps) having the lowest MSE of all steps, as represented in Panel **D**.

**Figure 2:**
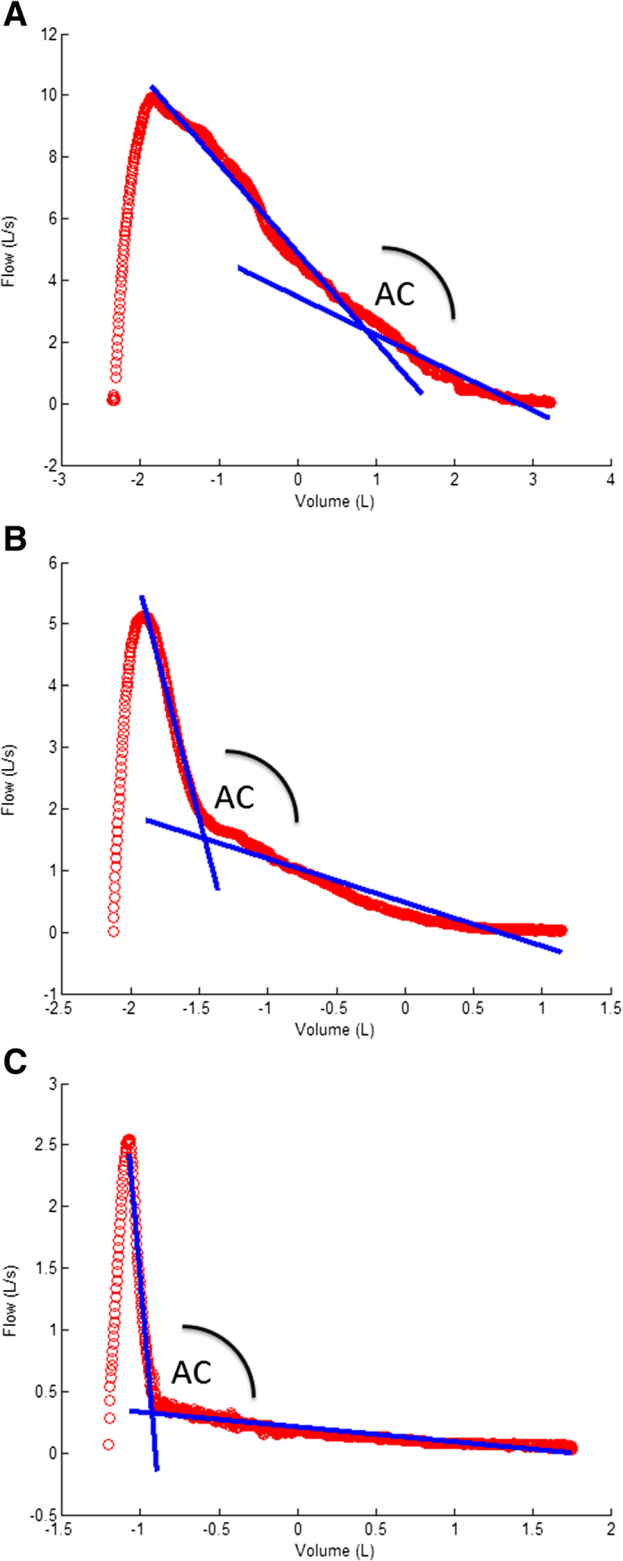
Examples of successfully calculated angles of collapse in different subjects: (A/ AC = 162° B/ AC = 133°C/ AC = 107°).

The algorithm for quantification of airway collapse computed the two best fitting regression lines on the expiratory phase of the flow-volume loop from peak flow to the end of expiration (Figure 1). The intersection of both best fitting regression lines determines the angular point of collapse with the first regression line representing the best fit of all data points from peak expiratory flow till collapse and the second line representing the best fit from collapse till end of FVC. To determine which couple of regression lines composes the best fit, a candidate reference point is created every 10 consecutive samples starting from peak flow till the end of expiration. In every step the MSE (mean square error) between the original data and the fitted lines is calculated with the lowest MSE designating the most appropriate reference point. Subsequently, pulmonary function tests with angles <90° and >180° are rejected, with remaining measurements ranging from 90° in case of maximal collapse to 180° when a perfect linear decrease of flow is present (Figure 2). Only 5.6% (N = 29) of patients had to be excluded either because AC quantification failed or was above the present limit of 180°. The main reason was a flow volume loop of suboptimal quality. In 2% (N = 10) of cases emphysema scores were not obtained from radiologists because of poor CT quality.

### Baseline characteristics

Baseline characteristics of 474 subjects with good quality measures of AC and visually assessed emphysema are presented in Table 1. Emphysema was present in 251 subjects of the total study sample. Mean (±SD) AC in subjects with emphysema was significantly lower 131° (±14°) compared to 152° (±10°) in subjects without emphysema, (p < 0.0001). As expected, the prevalence and the extent of emphysema was much lower in the group of subjects with normal FEV1/FVC ratio compared to subjects with a spirometry-based diagnosis of COPD (10% with median score of 2.1 (0.8-4.8) vs. 71% with a median score of 35 (8.3-58.5)). In non-COPD subjects AC was not significantly different between subjects with or without emphysema (157 ± 5 vs. 156 ± 7, p = 0.73). However, in COPD patients AC was significantly lower in patients with emphysema compared to the non-emphysema patients (130 ± 13 vs. 146 ± 11, p < 0.0001), even when stratifying according to former GOLD stages of COPD severity (Table 2). Severity of the disease was staged by FEV_1_ expressed as percent predicted as stated in former Global Initiative for Chronic Obstruction Lung Disease (GOLD) classification [28].

**Table 1:** Study population characteristics

	**No emphysema**	**Emphysema**
Patients, n	223	251
COPD, absent/present	124/99	14/237
Sex, M/F	178/45	192/59
Age, years	62 (58 - 67)	65 (59 - 74)
Smoking, pack yr.	40 (30 - 53)	48 (34 - 62)
BMI, kg/m^2^	27 (±4)	24 (±5)
FEV_1_, %predicted	91 (±25)	52 (±26)
FVC, %predicted	103 (±20)	88 (±24)
FEV_1_/FVC	0.68 (±0.11)	0.45 (±0.14)
K_CO_, %predicted	96 (±17)	69 (±21)
D_L,CO_, %predicted	82 (±17)	50 (±19)
Emphysema scores, %	0	32 (7 - 57)

Definition of *abbreviations*: BMI Body mass index, COPD Chronic obstructive pulmonary disease, D_L,CO_ Carbon monoxide diffusing capacity, F Female, FEV_1_ Forced expiratory volume in one second, FVC Forced vital capacity, K_CO_ Carbon monoxide transfer coefficient, M Male, Values are means ± SD; Emphysema score, age and smoking values are median and IQR.

**Table 2:** Angle of collapse in different GOLD stages

	**No Emphysema**		**Emphysema**		
	**N**	**AC**	**N**	**AC**	**p value**
No COPD	124	156 (±7)	14	157 (±5)	0.7261
GOLD I	48	152 (±6)	29	148 (±5)	0.0056
GOLD II	28	146 (±7)	73	138 (±9)	<.0001
GOLD III	16	137 (±6)	81	126 (±9)	<.0001
GOLD IV	7	123 (±16)	54	117 (±8)	0.0858

Values are means ± SD.

### Value of AC compared to pulmonary function variables

With univariate linear regression, similar relationships between emphysema scores and lung function variables (FEV_1_/FVC ratio, D_L,CO_, %predicted) were found as between emphysema scores and AC. Associations with FVC, %predicted and FEV1, %predicted were less pronounced (Table 3). Multivariate linear regression analysis revealed AC as best indicator of emphysema extent, not only in stepwise models taking only spirometry measurements into account (FVC, %predicted, FEV1, %predicted, FEV1/FVC), but also in models including both spirometry data and diffusing capacity (K_CO,_ %predicted, D_L,CO,_ %predicted) (Table 4). Logistic regression confirmed AC as significant predictor of emphysema presence whereas in a multivariate model, FEV_1_/FVC ratio seemed to be a better determinant (see Additional file 1: Tables S1 a/ and b/ in the online supplementary material). When restricting the multivariate logistic regression to the subgroup of patients with proven COPD, a significant relationship between emphysema and AC was confirmed, suggesting that FEV_1_/FVC has only superior associations with emphysema in the population without COPD.

**Table 3:** Relationship between functional variables and emphysema extent in univariate linear regression model

**Variables**	**R**^**2 **^**value**	**p value**
FVC, %predicted	0.1427	<.0001
FEV_1_, %predicted	0.4001	<.0001
FEV_1_/FVC	0.5030	<.0001
K_CO_, %predicted	0.4658	<.0001
D_L,CO_, %predicted	0.5065	<.0001
AC, degrees	0.5033	<.0001

R^2^ value represents variance explained by the model.

**Table 4:** Relationship between functional variables and visual CT-scores of emphysema by multivariate linear regression model with stepwise selection

**Variables**	**Partial R**^**2 **^**value**	**Model R**^**2 **^**value**	**p value**
1.			
AC, degrees	0.5032	0.5032	<.0001
FEV_1_/FVC	0.0211	0.5244	<.0001
2.			
AC, degrees	0.5056	0.5056	<.0001
K_CO_, %predicted	0.1137	0.6193	<.0001
FEV_1_, %predicted	0.0067	0.6260	0.0040

1/ Variables of spirometry (initial model: AC, FVC,%pred, FEV1,%pred, FEV1/FVC); 2/ Variables of spirometry and diffusion capacity (initial model: AC, FVC,%pred, FEV1,%pred, FEV_1_/FVC, D_L,CO_,%pred, K_CO_,%pred).

### Sensitivity and specificity of AC

**Figure 3:**
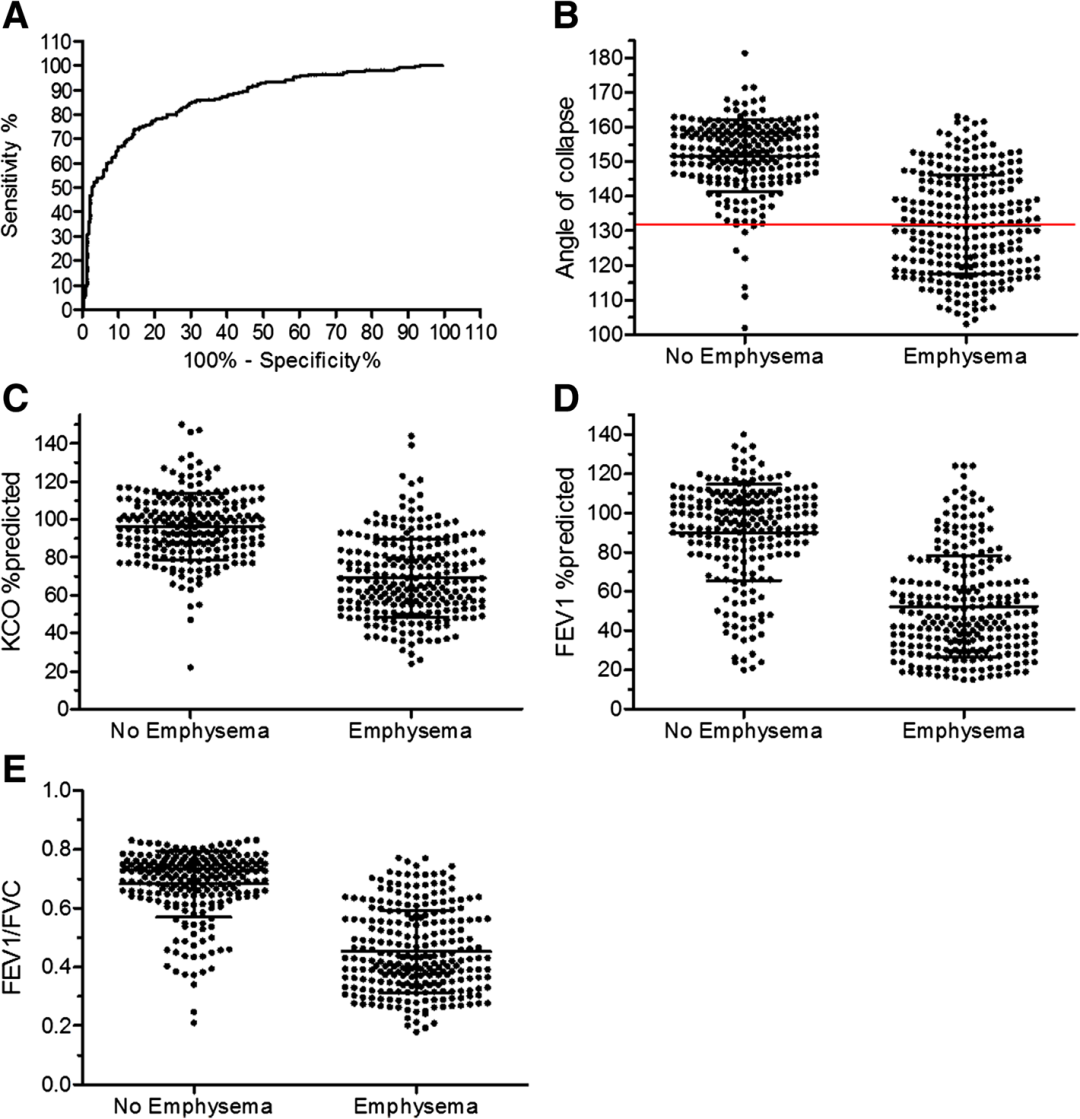
**Receiver operating characteristic (ROC) curve analysis.** Panel **A**/ ROC curve of AC to diagnose emphysema. Panels **B-E**/ Scatter graph of different variables within emphysema subjects and non-emphysema subjects. The horizontal line represents the best cut-off for sensitivity-specificity.

We used ROC curve analysis to compute the sensitivity and specificity of AC for predicting emphysema presence. An area under the curve of 0.863 (0.830-0.896) was found to be statistically significant (p < 0.0001). If using maximal sum of sensitivity and specificity to select best cut-off, ROC curve pointed to 143°, with specificity of 86% and sensitivity of 74%. However, if determining the cut-off based on the clinical need for high specificity and strong positive predictive value, ROC curve indicated 131° as most appropriate cut-off, with specificity of 97% and sensitivity of 51% (see Figure 3, panel A and B). An angle ≤131° resulted in a positive predictive value of 95.5% for the presence of emphysema. From the 133 subjects categorized as having emphysema based on the AC, only 6 were misclassified. A similar approach resulted in a 66% cut-off for K_CO_, %predicted and a 0.43 cut-off for the FEV_1_/FVC ratio with comparable results for specificity and sensitivity in predicting the presence of emphysema. When joining both cut-offs of either 131° for AC or 66%predicted for Kco, sensitivity rose to 67%, whilst positive predictive value and specificity remained within the same range (93% and 95%, respectively).

### Densitometric quantification of emphysema

Despite the heterogeneous acquisition of CT scans in our clinical cohort, we also calculated automated density scores based on Hounsefield Units (HU) to quantify emphysema. An additional number of 8 patients had to be excluded due to lack of density scores. When defining emphysema based on attenuation of at least 1% of the total lung volume below the -950 HU threshold, 446 (96%) subjects were categorised with emphysema. When using an arbitrary but more strict approach to define emphysema (>10% below -950 HU), only 213 patients were labelled with emphysema, which had also a best agreement with the obtained visual scores, kappa 0.393 (95%CI: 0.310 -0.476). Subsequent stratification according to the 10% cut-off demonstrated worse pulmonary function and significantly lower AC (134° ± 16° ) in the emphysema group compared to the controls (148° ± 13°) (p < 0.0001) (online Additional file 1: Table S2). Despite poor correlations between automated density scores expressed as proportion of voxels below the -950 HU threshold and pulmonary function variables (online Additional file 1: Table S3), multivariate linear regression still retained AC as best indicator of emphysema (Table 5). Multivariate logistic regression confirmed AC as significant and best predictor of emphysema (Online supplement, Additional file 1: Table S4).

**Table 5:** Relationship between functional variables and densitometric quantification of emphysema by multivariate linear regression model with stepwise selection

**Variables**	**Partial R**^**2 **^**value**	**Model R**^**2 **^**value**	**p value**
1.			
AC, degrees	0.1938	0.1938	<.0001
K_CO_, %predicted	0.0404	0.2342	<.0001
2.			
AC, degrees	0.1939	0.1939	<.0001
FEV_1_/FVC	0.0074	0.2013	0.0385
FEV_1_, %predicted	0.0083	0.2097	0.0280

1/ Variables of spirometry (initial model: AC, FVC,%pred, FEV1,%pred, FEV_1_/FVC); 2/ Variables of spirometry and diffusion capacity (initial model: AC, FVC,%pred, FEV1,%pred, FEV_1_/FVC, D_L,CO_,%pred, K_CO_,%pred).

When performing ROC curve analysis, and using high specificity and strong positive predictive value to select the best cut-off, ROC curve pointed to 124° but with an expected lower sensitivity of 67%, specificity of 91% and positive predictive value of 81% (Online supplement, Additional file 1: Figure S1).

### Validation study

Computation of airway collapse was repeated in a similar independent cohort in which successful quantification was present in 93.5% (N = 303) of subjects. 6.5% (N = 21) of subjects were excluded due to exceeded limit of 180° or failed quantification. Baseline characteristics are described in Additional file 1: Table S5 (online data supplement). Of note, emphysema based on visual scores was found in 182 (60%) of individuals and a significant difference in mean AC between emphysema and non-emphysema subjects, 129° ± 16° vs. 151° ± 11° (p < 0.0001), was confirmed. Furthermore, when 131°Cut-off for AC was applied, a specificity of 96% and sensitivity of 59% were found in the validation set. Positive predictive value remained very high (95.6%) with only 5 among 113 subjects being misclassified.

## Discussion

Our study demonstrates that airway collapse observed at forced expiration can be automatically quantified by a computer model as an angle varying from 180° to 90°, correlating with the presence and severity of emphysema. Although our method was not sensitive to identify mild emphysema in the early stages of smoke-induced lung disease, an angle ≤ 131° proved to be a reliable cut-off for the positive prediction of emphysema in smoking individuals.

Emphysema is characterized by the disruption of alveolar attachments leading to loss of alveolar-airway interdependence and reduced airway tethering during breathing [29]. Upon the generation of highly positive intra-thoracic pressures during forced expiration, airway collapse occurs. Since many years it is understood that collapse represented by a spirographic kink in expiratory flow volume loop is indicative for emphysema [20]. Recent studies elegantly confirmed reduced airway diameter on CT scan with the presence of emphysema [30,31]. To the best of our knowledge, our study is the first to validate the concept of emphysema-associated airflow collapse in a larger group of individuals comprising COPD patients of all severity stages as well as smoking controls. In our population, we found that collapse correlated well with severity of emphysema and was even better associated than measures of diffusing capacity in a multivariate approach, particularly in patients with established diagnosis of COPD based of FEV1/FVC ratio [32]. Despite the low sensitivity of our approach, the established cut-off of 131° yielded a positive predictive value of more than 95%. Revision of all 6 false positive cases revealed that 3 of them had alpha-1-antitrypsin deficiency resulting in panlobular emphysema. The latter may explain some misclassification on CT which would indicate an even higher accuracy [33,34]. Furthermore, when combining the cut-off of collapse with a potential cut-off of 66% for Kco, sensitivity improved to 67% for a similar specificity indicating that both measures still provided additional information for the prediction of emphysema [35].

An important strength of our approach is the use of a computer model to automatically quantify collapse. Previous studies have used a beta-angle for quantification [36]. With the latter method the angular point is fixed on the flow-volume curve at 50% of FVC, with one leg through peak flow and the other leg through the X axis at the end of expiration. A major disadvantage of such technique is that early collapse after peak flow is often underestimated whereas airflow limitation at the end of expiration is overestimated. Our methodology is different by the fact that our angular point is not obligatory located on the flow-volume loop but chosen as the intersection point of best fitting regression lines representative of the data. Such approach does not only provide a more exact quantification when collapse is difficult to visualize or even absent, it also results in a quantification that closely corresponds to visual estimates when the angle is obviously present. In this context a visual cut-off of 131° is clinically useful, especially for primary care physicians who don’t have standard access to CT scan or diffusing capacity. Indeed, the identification of emphysema with spirometry in a subgroup of smoking individuals in primary care, may envisage early referral for a more extended testing and, if needed, a more rigorous follow-up. In the past, other computational methods have been used to characterise airflow limitations on forced expiration [37]. One interesting approach, is the assessment of mean transit time (MTT) as a sensitive indicator of both large and small airways obstruction [38,39]. It is yet to be explored whether MTT is also useful in detection of emphysema at early stages and in more severe COPD [40].

As mild emphysema may also present without airflow limitation and collapse, one may hypothesize that the angle is rather representative of airway obstruction than of emphysema *per se*. It is obvious that the severity of emphysema is closely related to COPD severity, but the fact that within each former GOLD category patients with emphysema have significantly lower angles compared to their non-emphysematous counterparts, indicates that the angle quantifies flow limitation beyond FEV_1_. From a physiological point of view, airway obstruction must present with a curvilinear decrease of flow versus volume, whereas sudden drops in flow for little volume changes are representative of collapse [41]. Although AC is sensitive to collapse and closely relates to emphysema, we do not claim that AC is a unique characteristic of emphysema, as this also not the case for decreased diffusion capacity either. Moreover, future studies with dynamic CT scans are required to differentiate with dynamic collapse of the central airways in case of tracheobronchomalacia, often occurring in severe COPD [42].

Despite the fact that we confirmed our findings in a second independent cohort, our study has some limitations. Most importantly, we used validated semi-quantitative or visual scores for the characterization of emphysema because CT scans were obtained in clinical routine with different CT equipment, different acquisitions and variable use of intravenous contrast. When using automated density measures of emphysema (defined as the percentage of voxels below −950 Hounsfield Units (HU) at inspiration), we found the expected poor relationships between AC and emphysema percentage (R^2^ = 0.1948, p < 0.0001) [43]. Although we know that these relationships are not perfect even in larger cohorts with a uniform acquisition (e.g. in the COPD Gene study), correlations between automated scores and PFT measures usually reaches twice the value of ours [44,45]. Surprisingly, multivariate regression still retained AC as best indicator for emphysema determined on HU, which indicate that the observed relationships with AC are independent of the methodology to measure emphysema. Another limiting factor of this study is the incapability of the algorithm to correctly compute the best fitting regression lines in all cases. Nevertheless, when taking into account badly performed maneuvers, flow fluctuation and curvilinear flow volume loops, correct computation between 180° and 90° was possible in approximately 95% of individuals. Finally, our method failed in terms of sensitivity and is therefore less useful as screening tool for the early detection of emphysema. This occurrence is inevitable due the fact that airflow limitation may be absent in patients with early emphysema, often only appearing on CT scan [46]. Whether it is clinically relevant to be diagnosed at this early stage when airflow limitation and collapse are not yet present, remains to be explored.

Taken together, our data provide strong evidence that airway collapse is one of the best lung functional correlates of visually assessed emphysema on CT scan. In primary care, detection of emphysema on spirometry may identify a population at risk for clinical deterioration and specific follow-up.

## Abbreviations

AC: Angle of collapse; BMI: Body mass index; COPD: Chronic obstructive pulmonary disease; DL,CO: Carbon monoxide diffusing capacity; FEV1: Forced expiratory volume in one second; FVC: Forced vital capacity; HU: Hounsfield units; I CC: Intraclass correlation coefficient; KCO: Carbon monoxide transfer coefficient; MSE: Mean square error; MTT: Mean transit time; ROC: Receiver operating characteristic.

## Competing interests

The authors declare that they have no competing interests.

## Authors’ contributions

Study concept and design: MT, KJ, DB, MD, WJ; Analysis and interpretation of the data: MT, VE, AP, WJ; Radiology data interpretation and scoring: JC, WD, MH, PS; Writing of the manuscript: MT, MD, WJ; Critical revision of the manuscript. All authors read and approved the final manuscript.

## Supplementary Material

Additional file 1 Supplementary material. Click here for file
