# Supplementary material for: Computer quantification of airway collapse on forced expiration to predict the presence of emphysema
Source: Respir Res. 2013 Nov 19;14(1):131. doi: 10.1186/1465-9921-14-131 (PMC3870969; doi:10.1186/1465-9921-14-131)
Supplement: Additional file 1 — Supplementary material. [file 1465-9921-14-131-S1.doc]

**Supplementary material**

**Supplementary note S1:**

**Emphysema** **scores**: CT scans were obtained in a routine setting using different multi-detector row scanners with different acquisition parameters. All examinations were performed from the apex to the base of the lung. All patients were examined in supine position and the scans were conducted at e nd-inspiration.

**Visual examination:** Images were transferred to a PACS system (AGFA, Kontich, Belgium) where they were viewed on a monitor display system Coronis 2MP, resolution 1280x1200 (Barco, Kortrijk, Belgium) using standard lung window settings (1,500/-500 Hounsfield Units [HU]). Emphysema was semi-quantitatively assessed by a visual scoring system, due to heterogeneous acquisition induced by different scanners and various intravenous contrasts that were used. Scorings were obtained on thin section CT images at 1 mm collimation with sharp reconstruction filter (B70f) at three levels in both lungs. Level I or upper field was at the proximal 1/3 of the aortic arch, level II or middle field was at the tracheal carina and level III or lower field was just above the highest point of the higher hemi-diaphragm. A blinded radiologist specialized in thoracic imaging scored each of the CT scans for the presence and extent of emphysema. Emphysema was defined as an area of hypovascular low attenuation, graded at each level with an incremental 5% scale and averaged in a tissue score reflecting the extent of emphysema over both lungs. If emphysema was visually scored on any of the predefined fields, the patient was categorized as having emphysema.

**Densitometric quantification:** All CT scans were analysed using in-house developed algorithm. Segmentation of trachea, left and right lung was performed by a fully automated region growing program starting in the trachea and including all connected areas below -500 HU. In a second step, trachea and main bronchi were separated from the lungs, and segmented lungs were subjected to a noise reduction filter, since low dose protocol results in higher emphysema scores due to noise. The extent of emphysema was estimated using the percent of voxels with an apparent X-ray attenuation value below predefined density thresholds: areas with a X-ray attenuation below -950 HU (>19.1 ml/g lung tissue) were calculated and expressed as a percentage of the total lung volume ranging from 0%-100%. A cut-off value of ≥1% of total lung volume attenuated below -950 HU was arbitrarily chosen to be considered as abnormal and representing emphysema [21, 28]. Additionally, a more stringent approach with a cut-off value of ≥10% of total lung volume attenuated below -950 HU was used to define emphysema in a subsequent analysis.

**Table S1:**

**a:** Univariate logistic regression model with visually assessed emphysema expressed as a binary variable (present or absent) and an independent variables

| Variables | *OR (95% CI)* | p value |
| --- | --- | --- |
| FVC, %predicted | 0.969 (0.960 - 0.978) | <.0001 |
| FEV_1_, %predicted | 0.951 (0.943 - 0.959) | <.0001 |
| FEV_1_/FVC | 0.889 (0.871 - 0.906) | <.0001 |
| K_CO_, %predicted | 0.931 (0.919 - 0.943) | <.0001 |
| D_L,CO_, %predicted | 0.918 (0.904 - 0.932) | <.0001 |
| AC, degrees | 0.889 (0.870 - 0.908) | <.0001 |

Definition of abbreviations: OR – Odds Ratio, CI – Confidence Interval

**Table S1:**

**b/**Multivariate logistic regression model using stepwise selection with visually assessed emphysema expressed as a binary variable and all functional variables with: 1/All subjects, 2/ Subjects with COPD

| Variables | *OR (95% CI)* | p value |
| --- | --- | --- |
| 1. |  |  |
| FEV_1_/FVC | 0.922 (0.899 - 0.946) | <.0001 |
| K_CO_, %predicted | 0.966 (0.948 - 0.986) | 0.0007 |
| D_L,CO_, %predicted | 0.977 (0.955 - 1.000) | 0.0486 |
| 2. |  |  |
| AC, degrees | 0.939 (0.909 - 0.970) | <.0001 |
| K_CO_, %predicted | 0.964 (0.943 - 0.984) | <.0007 |
| D_L,CO_, %predicted | 0.971 (0.948 - 0.995) | 0.0192 |

**Table S2:** Population characteristics by densitometric quantitation of emphysema

|  | No emphysema | Emphysema |
| --- | --- | --- |
| Patients, n | 253 | 213 |
| COPD, absent/present | 105/148 | 34/179 |
| Sex, M/F | 198/55 | 166/47 |
| Age, years | 62 (58 - 69) | 64 (60 - 70) |
| Smoking, pack yr. | 43 (30 - 57) | 44 (31 - 60) |
| BMI, kg/m^2^ | 27 (±5) | 24 (±5) |
| FEV_1_, %predicted | 81 (±28) | 58 (±31) |
| FVC, %predicted | 99 (±21) | 92 (±25) |
| FEV_1_/FVC | 0.64 (±0.14) | 0.48 (±0.17) |
| K_CO_, %predicted | 92 (±19) | 70 (±22) |
| D_L,CO_, %predicted | 75 (±18) | 54 (±23) |
| Emphysema scores (% < -950HU), % | 4.4 (±2.7) | 28.6 (±13.4) |

Definition of abbreviations: BMI = body mass index; COPD = chronic obstructive pulmonary disease; D_L,CO_ = carbon monoxide diffusing capacity; F = female; FEV_1_= forced expiratory volume in one second; FVC = forced vital capacity; K_CO_ = carbon monoxide transfer coefficient; M = male;

Values are means ± SD; Age and smoking values are median and IQR;

**Table S3:** Pearson Correlation Coefficients (*R*) comparing density scores of emphysema (HU950) with PFT parameters

| Variables | R value | p value |
| --- | --- | --- |
| FVC, %predicted | -0.177 | <.0001 |
| FEV_1_, %predicted | -0.360 | <.0001 |
| FEV_1_/FVC | -0.438 | <.0001 |
| K_CO_, %predicted | -0.413 | <.0001 |
| D_L,CO_, %predicted | -0.402 | <.0001 |
| AC, degrees | -0.441 | <.0001 |

**Table S4**: Multivariate logistic regression model using stepwise selection with emphysema (>10% below -950 HU) expressed as a binary variable and all functional variables within whole dataset

| Variables | *OR (95% CI)* | p value |
| --- | --- | --- |
| AC, degrees | 0.958 (0.942 - 0.974) | <.0001 |
| K_CO_, %predicted | 0.967 (0.955 - 0.978) | 0.0007 |

Definition of abbreviations: OR – Odds Ratio, CI – Confidence Interval

**Table S5**: Validation population characteristics

|  | No emphysema | Emphysema |
| --- | --- | --- |
| Patients, n | 121 | 182 |
| COPD, absent/present | 63/58 | 12/170 |
| Sex, M/F | 94/27 | 132/50 |
| Age, years | 63 (59 - 70) | 62 (57 - 68) |
| Smoking, pack yr. | 42 (34 - 57) | 42 (30 - 56) |
| BMI, kg/m^2^ | 28 (±5) | 23 (±5) |
| FEV_1_, %predicted | 86 (±23) | 48 (±26) |
| FVC, %predicted | 102 (±19) | 84 (±23) |
| FEV_1_/FVC | 0.68 (±0.11) | 0.44 (±0.16) |
| K_CO_, %predicted | 92 (±18) | 62 (±21) |
| D_L,CO_, %predicted | 77 (±18) | 46 (±21) |
| Emphysema scores, % | 0 | 28 (5.8 - 55.2) |

Definition of abbreviations: BMI = body mass index; COPD = chronic obstructive pulmonary disease; D_L,CO_ = carbon monoxide diffusing capacity; F = female; FEV_1_= forced expiratory volume in one second; FVC = forced vital capacity; K_CO_ = carbon monoxide transfer coefficient; M = male;

Values are means ± SD; Emphysema score, age and smoking values are median and IQR;


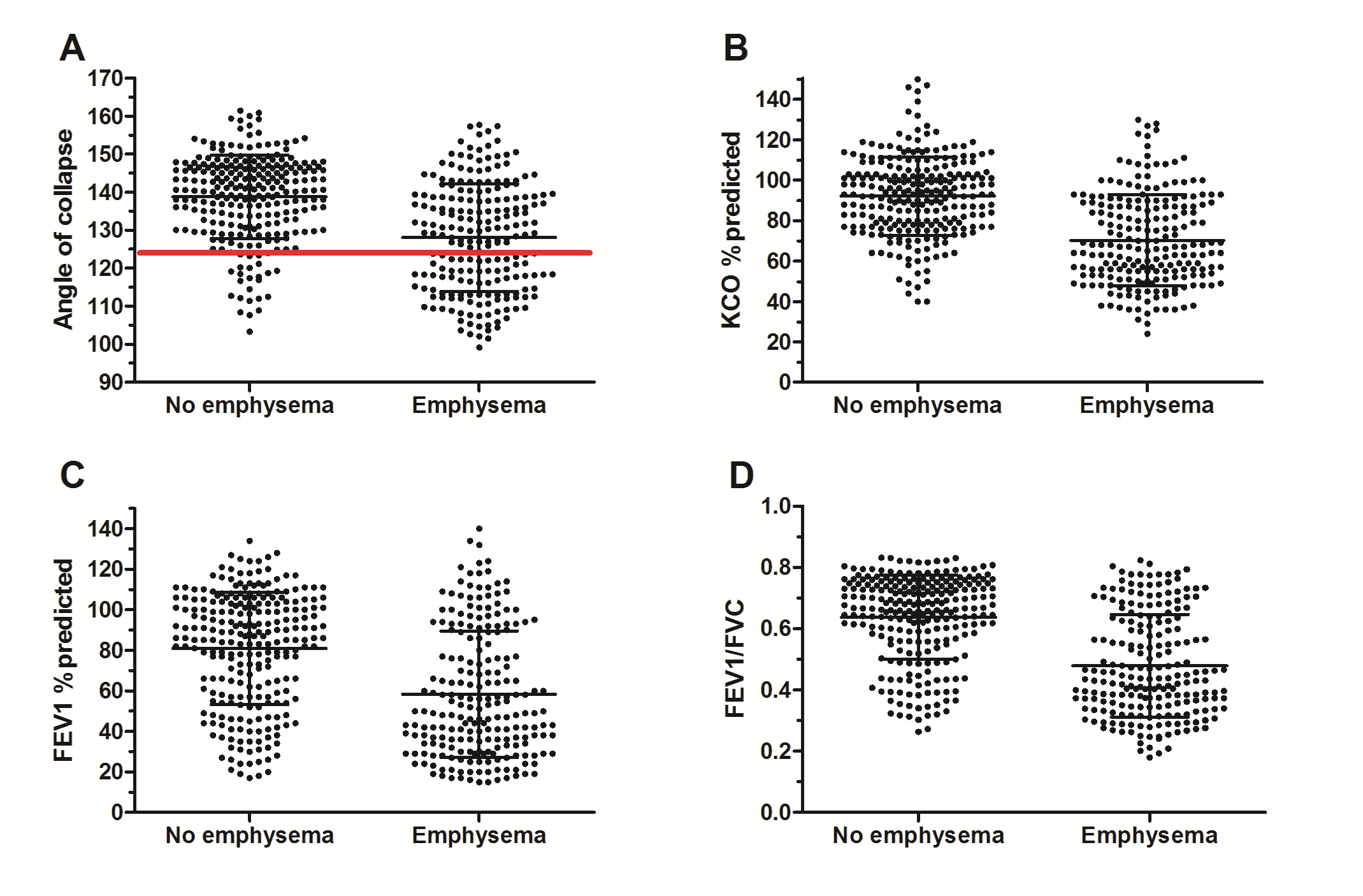


**Figure S1**

A- D/ Scatter graph of different variables within subjects with densitometric quantitation of emphysema and non-emphysema subjects. The horizontal line represents the best cut-off for sensitivity-specificity.
